# The Use of Dual-Mobility Implants Through a Direct Anterior Approach as a Safe and Effective Technique for Revision Hip Arthroplasty: The Two-Year Follow-Up of a Case Series

**DOI:** 10.7759/cureus.65680

**Published:** 2024-07-29

**Authors:** Jan Vanlommel, Mounir Cherkaoui, Dries Verrewaere, Victor Geenen, Anthony Van Eemeren, Maxence Vandekerckhove

**Affiliations:** 1 Orthopedics and Traumatology, AZ Sint-Lucas, Brugge, BEL

**Keywords:** revision, hip, arthroplasty, dislocation, anterior approach

## Abstract

Background

Dislocation is one of the most prominent and challenging complications following the revision of total hip arthroplasty (THA). Dual-mobility cups are an option to address this problem. There is, however, little data on the outcomes of modern modular dual-mobility (MDM) cups in the revision of THA. In this study, the clinical and radiological outcomes following the revision of THA with an MDM cup using the direct anterior approach (DAA) were evaluated.

Methodology

We retrospectively reviewed patients who underwent a revision of THA between March 2017 and July 2019. The inclusion criteria were a revision of THA using an MDM cup through the DAA. A uniform acetabular implant was used in each revision. Outcome measures were assessed radiographically and clinically. The clinical outcome measures consisted of dislocation, infection, and re-revision. Functional assessment was performed using the Harris Hip Score preoperatively and at the last clinical examination in our department.

Results

This study retrospectively identified a cohort of 26 patients who underwent a revision of THA. Two patients were excluded due to incomplete follow-up because they died. Finally, 24 patients were included. A total of 17 isolated acetabular revisions and seven complete revisions were performed with a mean follow-up of 39 months (range = 29-59). No dislocations or deep infections were observed in our population to date. Except for one case of early aseptic loosening of the acetabular component, we observed no other signs of loosening, osteolysis, migration, or intraprosthetic dislocation.

Conclusions

THA revision through the DAA using an MDM cup is a safe and effective procedure. We observed no dislocation in a high-risk population undergoing THA revision surgery during a minimal follow-up of two years.

## Introduction

The revision rate of total hip arthroplasty (THA) has grown steadily with the increasing number of primary THA over the last few years. The reported dislocation rate after revision THA ranges from 5% to 39%, two to five times higher than after primary THA. Some concepts have been introduced to reduce complications after revision surgery, but efficiency and implant stability remain the major concerns [1-6]. These include abductor repairs [7], increasing hip offset [7,8], using a different surgical approach [2] or different implants, such as larger diameter femoral heads, constrained liners, or, more recently, dual-mobility (DM) constructs [7-10].

The DM concept was introduced in the seventies in an attempt to reduce dislocation following THA [8] and to avoid complications occurring with other implants [11]. Specifically, large femoral heads raised concerns regarding accelerated wear and a higher incidence of adverse local tissue reaction (ALTR) and constrained liners encountered high rates of mechanic failure, dislocations, and movement restrictions [11].

In DM constructs, the acetabular component articulates with a head out of polyethylene (PE), which encloses a smaller metal or ceramic 28 mm head. The highly polished articular surface of the acetabular component may be the inner surface of a monoblock acetabular shell (standard DM) or, in the case of modular DM (MDM), a separate modular component made of cobalt-chrome inside a modular acetabular cup in titanium [9]. This design provides motion between the 28 mm ceramic or metal femoral head and the inner concave surface of the PE bearing, as well as between the PE bearing and the inner surface of the metallic acetabular cup when a larger range of motion is required [12]. As a result, it merges two concepts by maximizing the size difference between the outer cup and inner ball, i.e., Charnley’s principle of low frictional torque arthroplasty, while also maximizing the head-neck ratio to reduce the risk of impingement and increase the jump distance [8]. The ability to initially fixate the shell with a screw, which is usually preferred in the revision setting, is an advantage the MDM cup has over the standard DM cup [10]. Dual mobility adds stability without restriction, potential impingement, and stress transfer and therefore lowers the risk of aseptic loosening in comparison to constrained liners [9]. In the context of revision THA, however, there is relatively little data on the outcomes of MDM cups [10].

The potential efficacy of DM cups in preventing or treating instability after revision THA can be diminished if the abductor mechanism is compromised [13]. Therefore, we can assume it is essential to preserve the abductor apparatus during primary and revision THA. According to some studies, the direct anterior approach (DAA) is associated with improved dynamic hip stability and a decreased risk of hip dislocation after surgery because it follows an intermuscular and internervous plane with no risk of denervation of the hip abductor muscles [14].

Because abductor muscle deficiency is a factor contributing to instability and dislocation after revision surgery, we can hypothesize that the DAA, which preserves the abductor apparatus, is a valid alternative to the other conventional surgical approaches [1].

Yun et al. reported that refractory hip instability in THA may be effectively managed with an MDM articulation through the DAA in a group of 15 patients [15].

This retrospective study aimed to investigate the risks of dislocation after revision THA, for various reasons, using a DM construct through the DAA. We also investigated postoperative implant survival, cup migration, loosening, and osteolysis.

## Materials and methods

**Figure 1:**
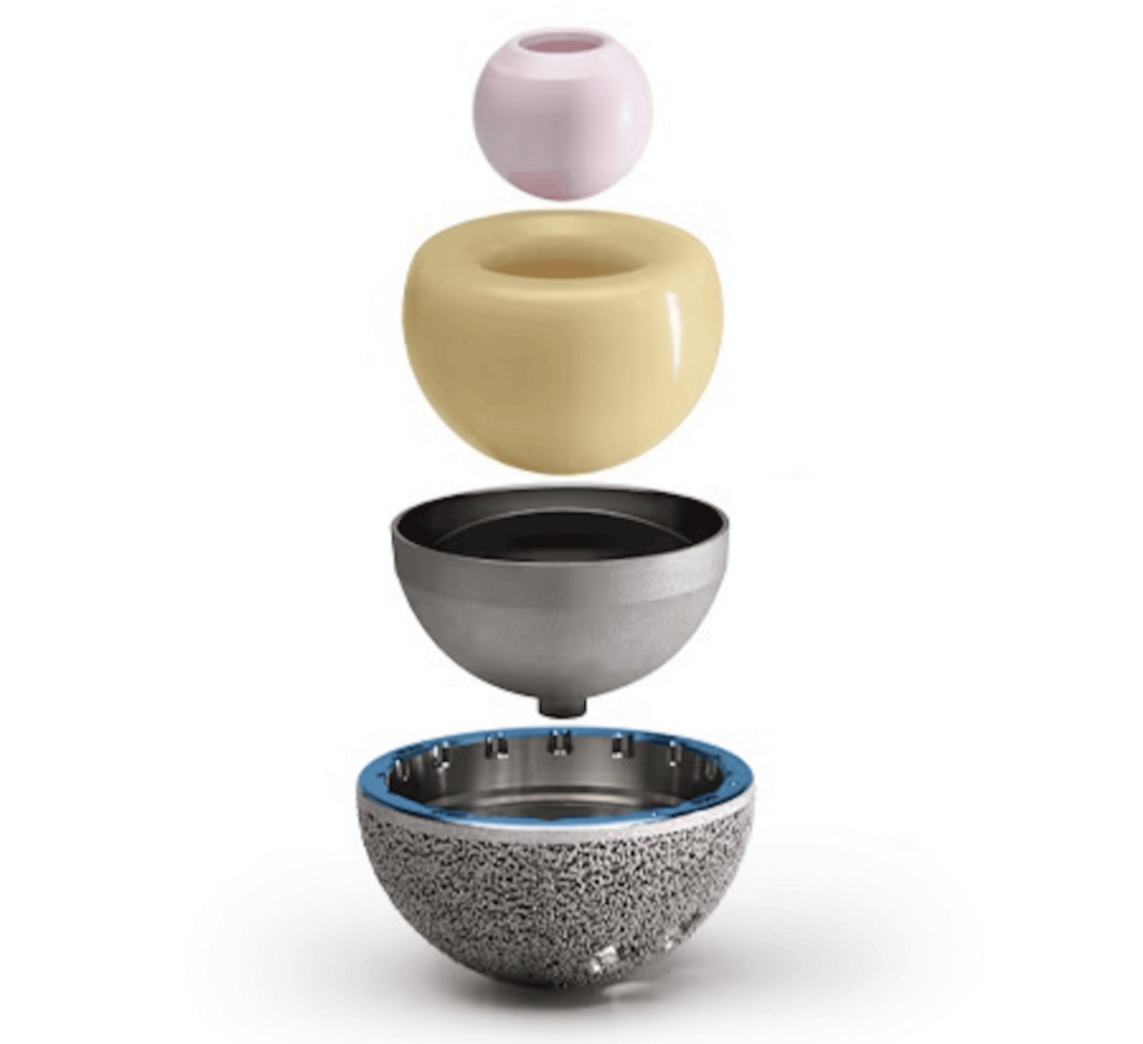
Disassembled modular dual-mobility construct.

**Figure 2:**
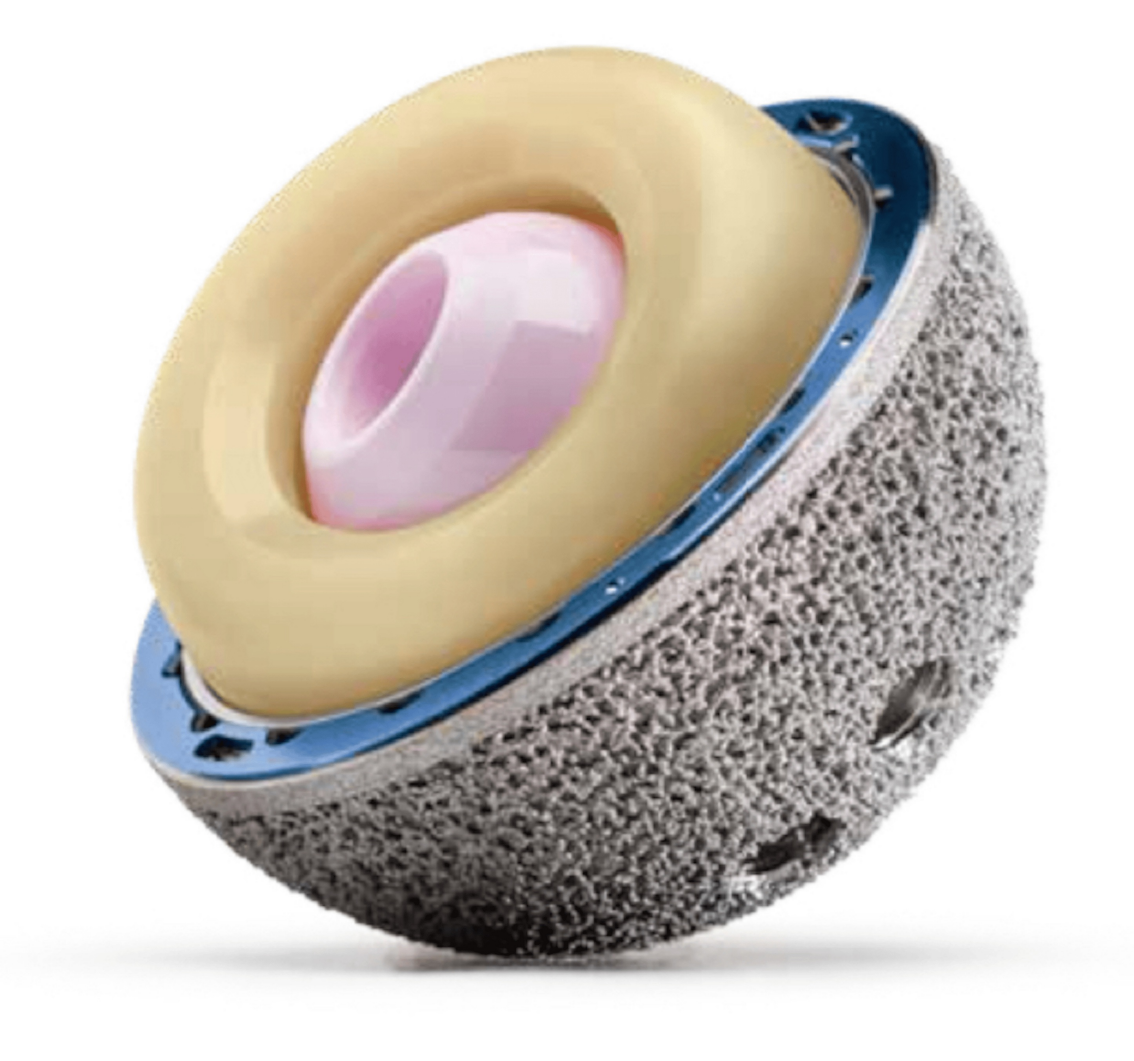
Assembled modular dual-mobility construct.

This study was approved by the Ethics and Institutional Review Board of AZ Sint-Lucas, Brugge, Belgium (approval number: 140). We retrospectively reviewed patients who underwent revision THA between March 2017 and July 2019. The inclusion criteria were revision THA using an MDM cup through the DAA. A uniform acetabular implant (G7 Dual Mobility Construct, Zimmer Biomet, Warsaw, IN, US) was used in all cases (Figures 1, 2).

A single senior surgeon (JV) with experience in DAA performed all revision procedures through the DAA. Ceramic or metal heads (28 mm) were used depending on the biological age of the patients. The femoral head is articulated with a vitamin-E-infused PE bearing. A standard surgical table with the possibility of hyperextension was used for all procedures. Patients were placed in the supine position, scrubbed from the umbilicus down to both feet, and the operation zone was draped. Leg length could be visually evaluated perioperatively. A classic longitudinal skin incision was used, starting approximately 3 cm laterally and 3 cm distally from the anterior superior iliac spine aiming toward the fibular head. This was preferred over a “bikini” incision because the longitudinal incision is easier to extend.

After superficial subcutaneous dissection, the fascia of the tensor fascia lata (TFL) was incised longitudinally, and deeper dissection was performed within this fascial sheath to prevent direct damage to the lateral femoral cutaneous nerve (LFCN). The intervals between the sartorius muscle and TFL and the rectus femoris muscle and TFL, respectively, were prepared by blunt dissection. After coagulating/ligating the ascending branches of the lateral circumflex artery, the hip capsule was exposed anterolaterally by mobilizing the gluteus minimus and TFL laterally and the iliocapsularis and rectus femoris medially. A subtotal capsulectomy was performed next. Subsequently, the femoral head was gently removed from the femoral component, carefully avoiding damage to the taper. After femoral head removal, the lateral capsule was released from the gluteus minimus and resected. To complete the mobilization of the proximal femur, the capsule on the inner side of the lateral trochanter was released until the internal obturator tendon was exposed. The acetabular component was removed, and sequential reaming was performed. Component stability was assessed with a trial component and the correct positioning was confirmed with intraoperative fluoroscopy.

When femoral revision was performed, a special double-pronged retractor was placed around the greater trochanter, and the leg was adducted and externally rotated (figure-four position in adduction with the surgically treated leg under the untreated leg). In the case of a well-fixed cementless stem, if necessary, an osteotomy, as described by Thaler et al., was performed [16]. In the case of a cemented component, the cement was removed stepwise. If necessary, extra releases were performed. Subsequently, gradual reaming was performed, and a femoral component, cemented if necessary, was placed.

**Figure 3:**
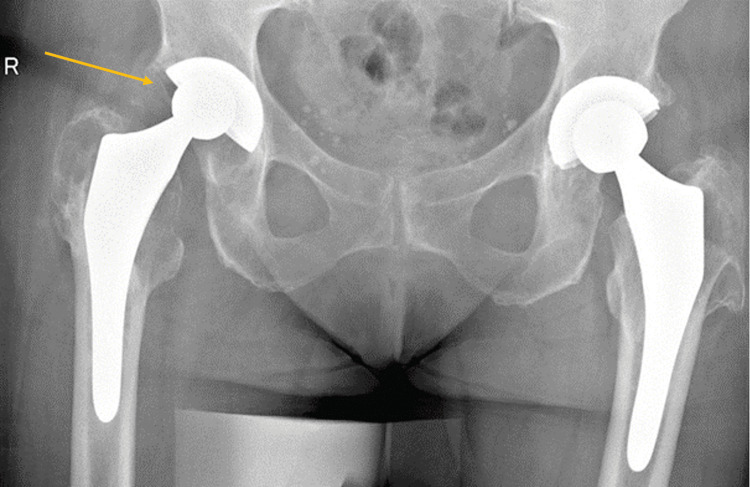
Anteroposterior pelvic X-ray of an 84-year-old patient with polyethylene wear 18 years after right primary total hip arthroplasty performed through the direct lateral approach. Bone scintigraphy revealed loosening of the femoral component.

**Figure 4:**
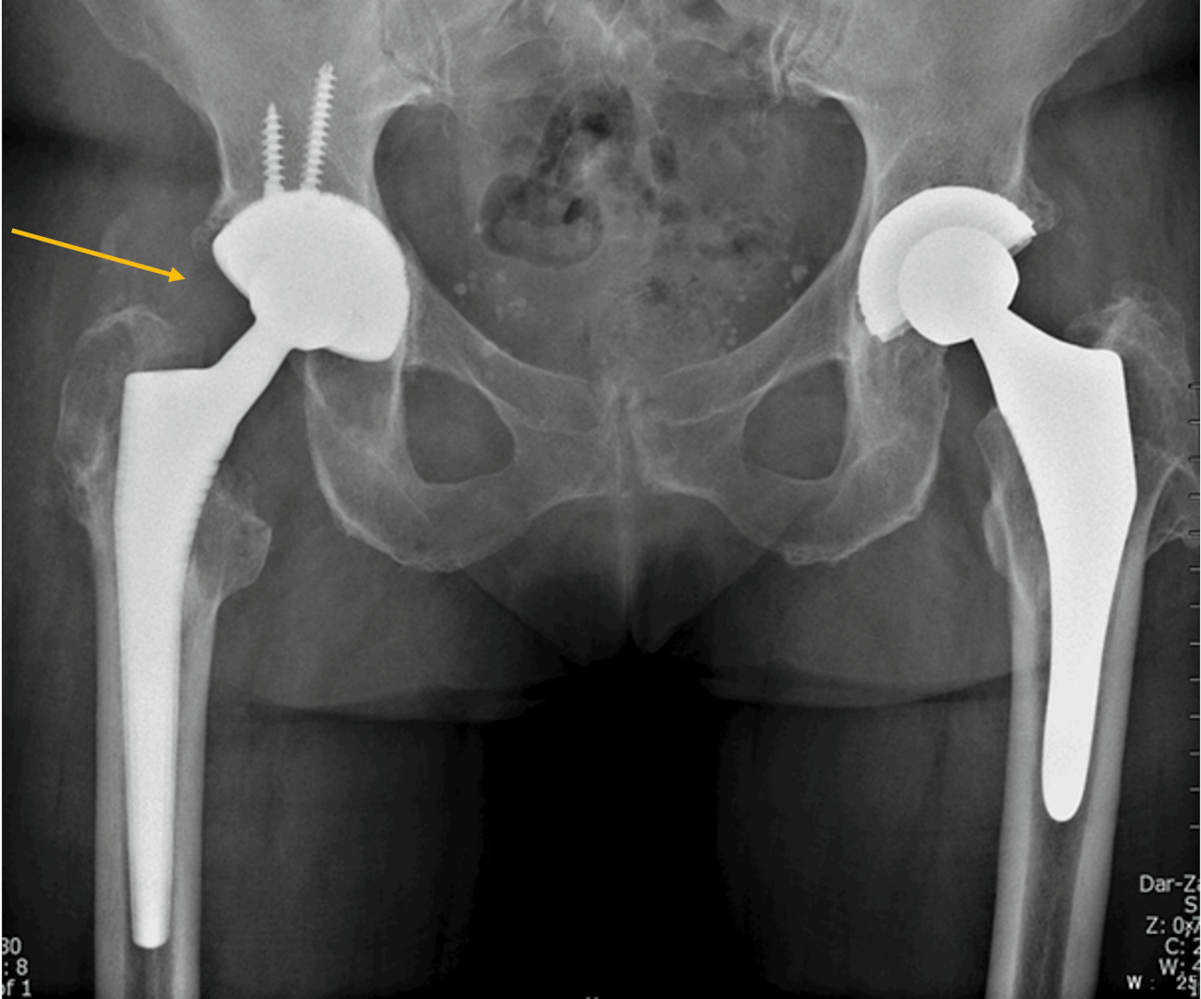
Anteroposterior pelvic X-ray six weeks after right revision total hip arthroplasty using a dual-mobility construct and a cementless stem.

**Figure 5:**
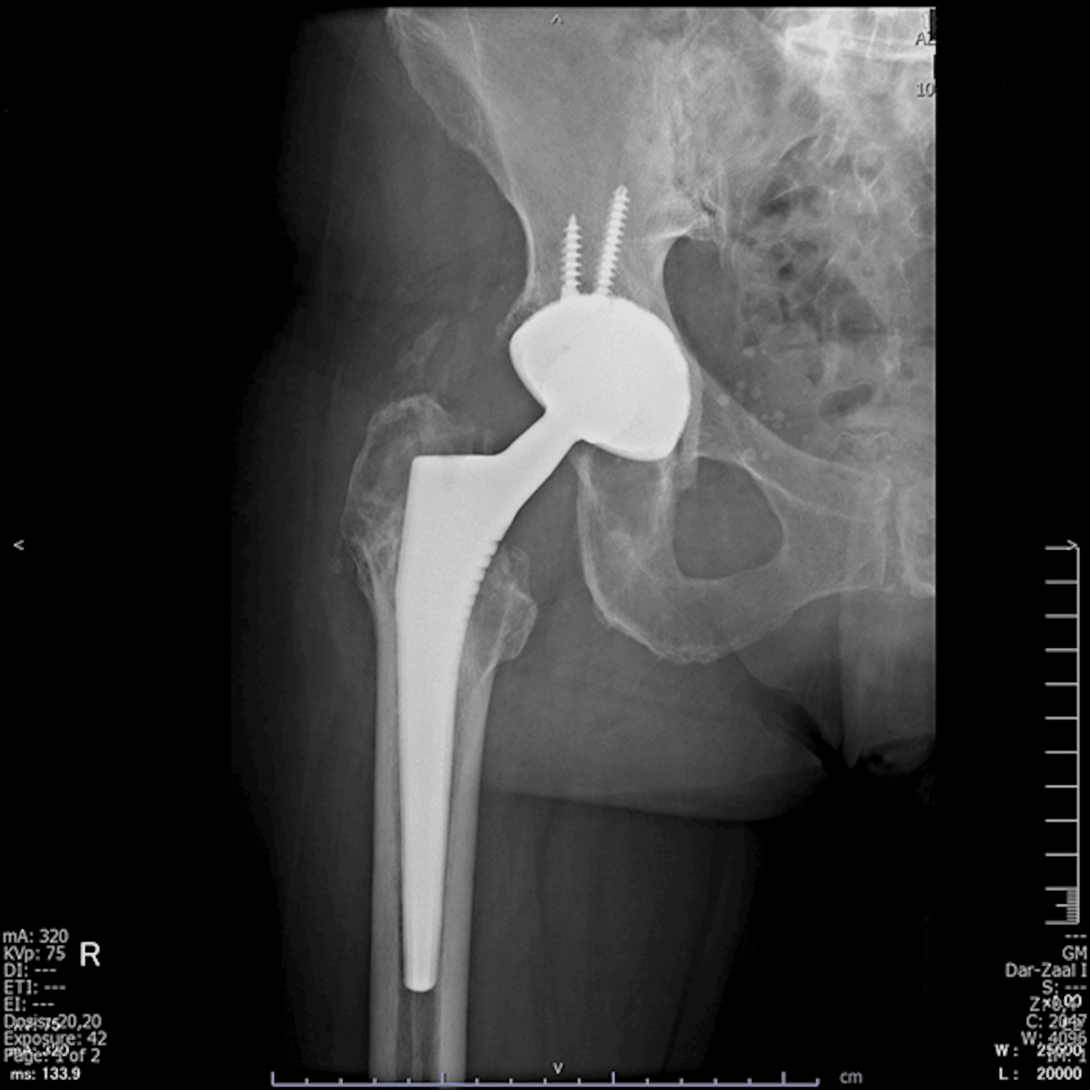
Anteroposterior right hip X-ray of the same patient one year after revision surgery.

Postoperatively, patients were evaluated clinically and radiographically at regular intervals, particularly at two weeks, six weeks, three months, and one year after surgery, and thereafter annually. Full weight bearing was allowed on the first postoperative day, though crutches were advised for six weeks. Outcome measures were assessed radiographically and clinically. Radiological follow-up comprised regular standardized anteroposterior radiographs of the pelvis and lateral views of the hip (Figures 3-5). On these X-rays, attention was paid to signs of intraprosthetic dislocation (IPD), loosening, migration, or osteolysis.

The inclination angle of the cup, measured as the angle between the inter-tear drop line and a line through the long axis of the cup on anteroposterior radiographs, and leg length discrepancies were also measured. Acetabular defects were classified using the Paprosky classification [17]. Clinical outcome measures consisted of dislocation, infection, and re-revision. Functional assessment was performed using the Harris Hip Score (HHS) preoperatively and at the last clinical examination in our department.

## Results

We researched 26 patients from the hospital database. Among them, two patients died (for reasons not related to surgery) and were excluded due to incomplete follow-up. Finally, this study included 24 patients (6 male and 18 female). The mean age at surgery was 77.1 years (range = 47-90 years). The mean body mass index was 27.1 kg/m² (range = 20.3-38.1 kg/m²). Patient demographics and postoperative data are listed in Table 1.

**Table 1:** Patient demographics and postoperative data. BMI: body mass index; LLD: leg length discrepancy

Patient demographics/Postoperative data	Value	Range
Gender (male/female)	6/18	-
Mean age (years)	77.1	47–90
Mean BMI (kg/m^2^)	27.1	20.3–38.1
Isolated acetabular revision/Complete revision	17/7	-
Mean operating time (minutes)	122	83–188
Mean follow-up (months)	15	6–26
Mean cup inclination (degrees)	42.1	32.9–50
Mean LLD (mm)	-1.33	-12–+8

The initial surgical approach of the 24 patients consisted of six direct lateral, 15 posterolateral, and three DAAs. Based on Paprosky’s classification, there were seven grade I, four grade IIA, two grade IIB, five grade IIC, three grade IIIA, and three grade IIIB (Table 2) [17].

**Table 2:** Paprosky classification of acetabular defects.

Paprosky classification	Number of cases
Grade I	7
Grade IIA	4
Grade IIB	2
Grade IIC	5
Grade IIIA	3
Grade IIIB	3

There were 17 isolated cup revisions and seven complete (femur and acetabular) revisions performed. In four cases, the revised stem was cemented. The mean surgical time was 122 minutes (range = 83-188 minutes). The indications for revision were aseptic loosening (n = 15), recurrent dislocation (n = 5), periprosthetic fracture (n = 3), and ALTR (n = 1) (Table 3).

**Table 3:** Indications for revision of total hip arthroplasty.

Indications for revision	Number of cases
Aseptic loosening	15
Recurrent dislocation	5
Periprosthetic fracture	3
Adverse local tissue reaction	1

**Figure 6:**
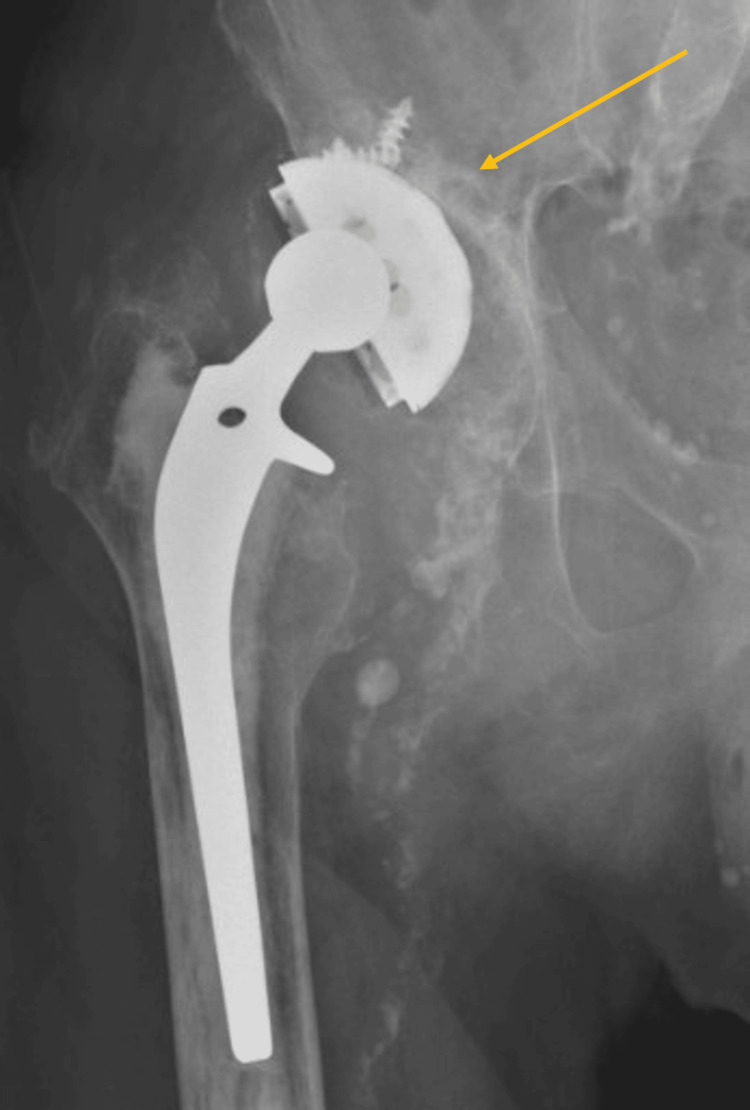
Anteroposterior pelvic X-ray of an 88-year-old patient with aseptic loosening and migration of the acetabular component (grade IIIB Paprosky acetabular defect).

**Figure 7:**
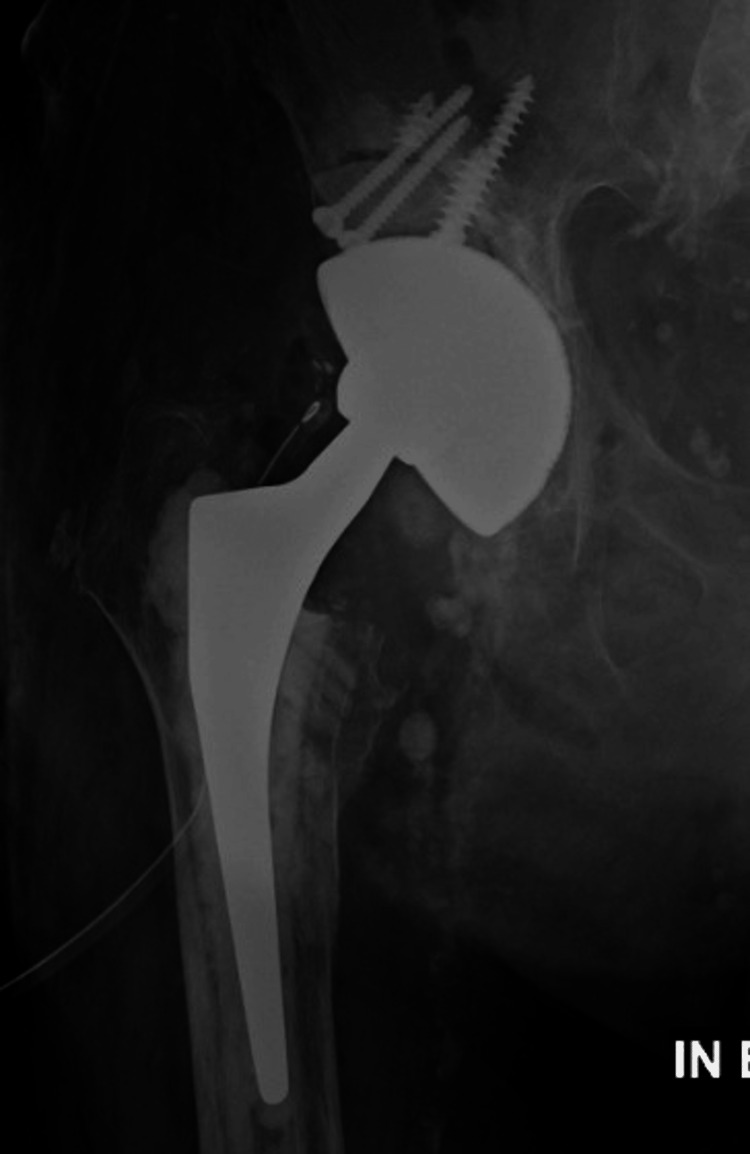
Postoperative X-ray three months after cup revision through the direct anterior approach.

**Figure 8:**
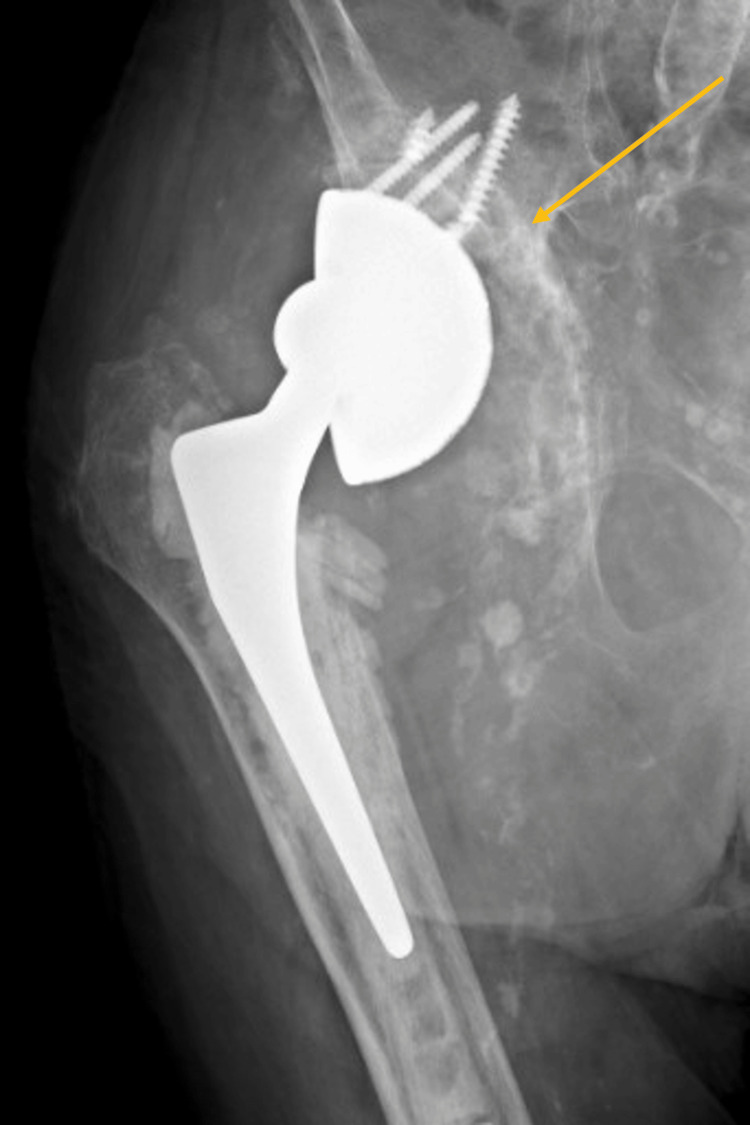
Anteroposterior pelvic X-ray showing early loosening of the revised acetabular component.

**Figure 9:**
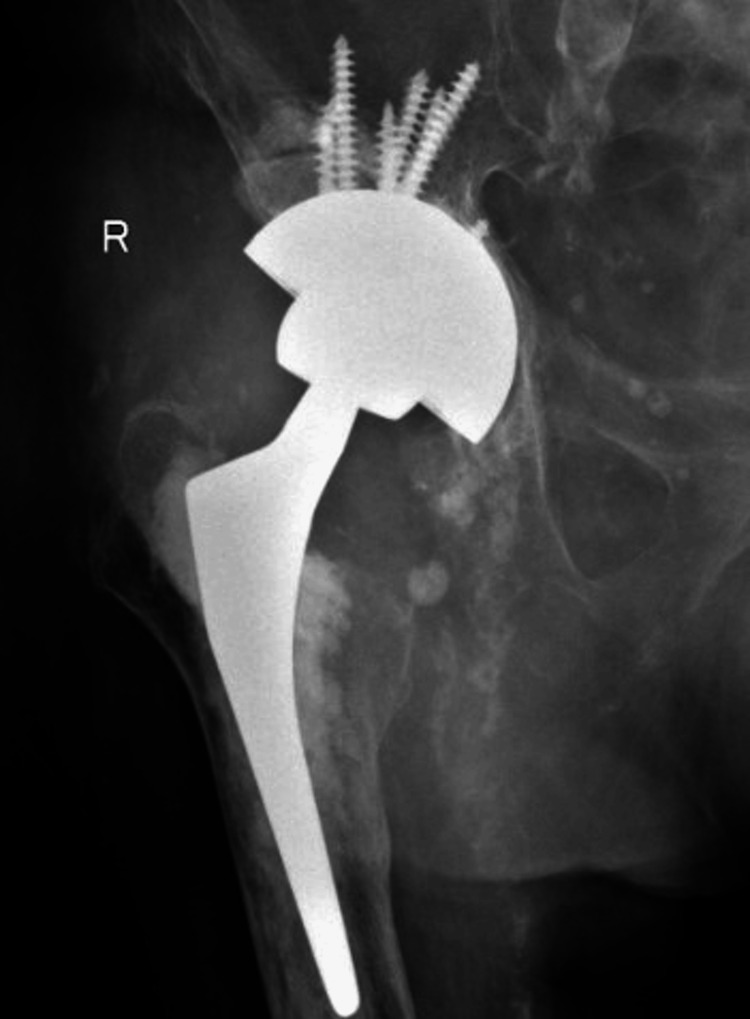
One year after the re-revision of the acetabular component through the posterolateral approach. No signs of loosening can be noted.

Regarding the outcome, we have observed no dislocations in our population to date. One patient experienced a superficial wound infection, which was treated conservatively with antibiotics and required no revision surgery. We did not encounter deep infections or deep vein thrombosis. At the final follow-up, no LFCN injury was observed. At the final follow-up, the mean HHS was 87.1 points (range = 83-94, SD = 3.99), whereas it was 55.7 points (range = 44-74, SD = 7.65) preoperatively (p < 0.001). All-cause survivorship in our study was 96.2%, as one patient experienced early loosening and migration of the acetabular component, which required re-revision. This patient was an 88-year-old male with a body mass index of 24.5 kg/m² who underwent a primary THA in 1993. The indication for revision surgery was an aseptic loosening of the primary acetabular component, with a grade IIIB Paprosky acetabular defect (Figure 6). A complete revision was performed using a cemented stem and a cementless cup secured with screws. The postoperative course was uneventful, and the patient had no complaints at the three-month follow-up (Figure 7). After four months, however, the patient presented with recurrent, progressive pain and a limp. Early loosening and migration of the cup were seen on radiographic imaging at this time (Figure 8). A re-revision was performed through the posterolateral approach and posterior structural allografts were applied. The cup was secured with screws as well. The patient experienced no other complications or adverse effects at this time (Figure 9). All the complications are listed in Table 4.

**Table 4:** Complications during follow-up.

Complications	Number of cases
Deep vein thrombosis	0
Dislocation/Intraprosthetic dislocation	0
Superficial infection	1
Deep infection	0
Acetabular component loosening and migration	1
Femoral component loosening	0
Nerve injury	0
Total complications	2

On radiological examination, we observed no loosening or migration of a single femoral component, and there were no signs of osteolysis or IPD. The mean cup inclination was 42.1° (range = 32.9° to 50°). The mean leg length discrepancy was -1.33 mm (range = -12 mm to +8 mm).

## Discussion

In this study, the clinical and radiological results of revision THA with an MDM cup using the DAA were reviewed. We observed no dislocations or IPDs during follow-up. There was one case of early loosening of the acetabular component. Therefore, one re-revision procedure was performed, resulting in a survival rate of 94.7%. Further complications in our study included one superficial infection, which could be treated conservatively without the need for surgery.

Regarding survivorship, this study showed similar survival rates in comparison with the reported literature, ranging between 91% and 97.7% [4,5,10,11]. In the systematic review by Levin et al. [11], several studies showed higher all-cause and aseptic survivorship for DM cups compared to fixed-bearing prostheses. A meta-analysis of the pooled data showed a trend toward favoring DM cups regarding aseptic and all-cause survivorship, but the difference was not statistically significant.

Within the available literature, there are several indications for the revision of THA. These include loosening, infection, periprosthetic fractures, ALTR, and component malposition [1,2,8,18]. Dislocation and recurrent instability remain among the most prevalent indications for both revision and re-revision surgery [11]. Both have a multifactorial etiology, which includes revision surgery itself, as this carries an inherently higher risk for dislocation as well as recurrent instability [11,19]. Risk factors can be divided into patient-related and procedure-related factors. Examples of procedure-related factors are the revision of a single component, such as PE liner exchange, the use of a small diameter femoral head, or the use of a standard rim liner [8,19]. Patient-related factors contributing to recurrent instability include age, obesity, the number of previous revision surgeries, a history of instability before revision surgery, abductor muscle deficiency, trochanteric non-union, femoral head osteonecrosis, and severe acetabular and femoral bone loss [2,19].

The reported prevalence of dislocation in revision THA varies from 5% to 39% [4-6]. In a recent systematic review by Levin et al. evaluating the performance of newer-generation DM cups in a revision setting, the dislocation rate was 2.2%, which is in line with the results of our current study showing a dislocation rate of 0% [11]. Their meta-analysis revealed significantly lower odds of dislocation (odds ratio = 0.24, p = 0.002) for DM cups compared to fixed-bearing prostheses [11]. This supports the belief that DM cups render greater postoperative stability in patients undergoing revision THA [20-23]. Regarding the unique risk of IPD, our study showed a prevalence of 0%, which is comparable to the prevalence reported in the literature varying between 0% and 3.7% [6,11,23]. However, IPD typically occurs several years after implantation, so longer-term follow-up will be necessary to assess if this low risk of IPD persists in these newer DM cups [24].

The DAA has been gaining popularity over the last few years as a minimally invasive approach through an intermuscular and internervous plane. Particularly in the context of complex arthroplasty problems, the DAA, which is associated with less soft tissue trauma [14], is a valuable approach to preserving muscular tissues that have already been compromised by previous surgery [21]. In a study by Hailer et al. with data extracted from the Swedish Hip Arthroplasty Register, the posterior approach was associated with a higher risk of dislocation in comparison to the direct anterior and direct lateral approaches [22]. However, there are currently no specific studies concerning the risk of dislocation after revision THA secondary to the used approach [17]. Therefore, it is impossible to confirm which approach is more effective for revision THA, specifically concerning the risk of dislocation [2].

Regarding functional recovery, there is a slight tendency to favor the DAA. Some studies report improved strength, healing, and proprioception in the direct postoperative period after the DAA and faster recovery of function and gait ability when compared to the posterior approach [1]. According to a study by Cogan et al., the functional results after the DAA are better in comparison to the direct lateral approach up to one year postoperatively, but this difference evens out two years after surgery [2]. A recent study by Tamaki et al. argues that early functional recovery after surgery through the DAA is comparable to other approaches [20]. We conclude that there is no consensus in the literature comparing different approaches for revision surgery regarding functional recovery and that more evidence is needed on this topic to make sensible conclusions. Yun et al. reported that refractory hip instability in THA may be effectively managed with an MDM articulation through the DAA in a group of 15 patients [15].

The DAA has several technical advantages in the setting of revision of THA. It provides easy access to the acetabulum with good visualization without detaching the abductor apparatus [20]. In the case of an isolated acetabular procedure, the exposure is equal to that of a primary procedure, so postoperative recovery and rehabilitation, including weight-bearing, are similar to after a primary THA [21]. Besides, when performing revision THA in a patient with a former posterior or anterolateral approach, there is usually less scarring [21].

Potential advantages of MDM cups include increased implant stability, jump distance, and range of motion, as well as improved load dispersion [7,12]. Another advantage is the potential ability to reduce a dislocated DM component without the need for open surgery [7]. Early concerns such as excessive PE wear and increased risk of aseptic loosening, in combination with the unique risk of IPD initially limited its widespread use [11]. However, newer-generation DM cups have improved designs that reduce these complications and show promising results regarding dislocation rates, survivorship, and complications [10,11].

Several studies have reported concerns about the release of metal ions following the implantation of a DM cup. Furthermore, adding an extra cobalt-chrome interface by adding modularity in MDM cups has raised these concerns even more [9,10,18,25]. There is limited data about DM cup implantation and metal ion release, and the available literature is inconclusive. In revision THA due to failed metal-on-metal prostheses and taper corrosion, using DM implants may yield more stability, particularly when soft tissue destruction is present, and a more stable implant is indicated [11]. Besides, the DAA provides optimal exposure to the acetabulum, allowing ideal cup positioning, which is a determinant factor of in vivo wear [14,18]. The topic remains controversial, though, with further research needed to confirm that the advantages of MDM cups outweigh the risks regarding metal ion release following implantation [11]. The consensus, however, which we follow, is that MDM cups should be reserved for high-risk situations, such as older or obese patients, patients suffering from psychological disorders, patients with an abductor deficiency, or patients suffering from neuromuscular disorders [11,18,25]. In younger patients with a longer life expectancy, ceramic-on-ceramic (CoC) bearings might be an attractive option to overcome these potential complications associated with MDM cups. Potential advantages of these modern bearings are biocompatibility, wear rate, and lubrication properties, but prospectively designed or randomized studies are lacking and needed to confirm CoC as an optimal solution for revision THA cases. Moreover, CoC bearings have unique potential adverse effects, such as squeaking and ceramic fractures [26].

We acknowledge several limitations to our current study. First, the duration of the follow-up was relatively short. This is less relevant regarding dislocations, as reported data suggests that most dislocations occur within three months after surgery [10,11]. Concerning serum metal ion levels and loosening, however, this raises more problems as these complications develop later. A future study with an extended follow-up could provide valuable insights into the longevity and durability of these implants. Second, our study has a retrospective design, which is more prone to bias in comparison to prospective study designs. This design may also affect the accuracy of outcomes. Another limitation of our study is the small study population. This, however, is a limitation currently inherent to this topic. Finally, we did not include a control group comparing results with revision THA performed through another approach. Further evidence is needed to evaluate the outcomes after revision THA through DAA compared to other approaches.

## Conclusions

Performing revision THA with an MDM cup using the DAA is a safe and effective technique with a very low dislocation rate, though it should be reserved for patients with an increased risk for instability. Further research is needed to address the remaining controversy about metal ion release and ALTR, as well as studies with longer-term follow-up to further evaluate the incidence of migration, loosening, and osteolysis following implantation of an MDM construct.
